# The Gut Microbiome Is Altered in Postmenopausal Women With Osteoporosis and Osteopenia

**DOI:** 10.1002/jbm4.10452

**Published:** 2021-01-19

**Authors:** Elizabeth A Rettedal, Bolaji L Ilesanmi‐Oyelere, Nicole C Roy, Jane Coad, Marlena C Kruger

**Affiliations:** ^1^ Food Nutrition & Health Team AgResearch Grasslands Palmerston North New Zealand; ^2^ Riddet Institute Massey University Palmerston North New Zealand; ^3^ School of Health Sciences College of Health, Massey University Palmerston North New Zealand; ^4^ Department of Human Nutrition University of Otago Dunedin New Zealand; ^5^ High‐Value Nutrition National Science Challenge Auckland New Zealand; ^6^ Liggins Institute University of Auckland Auckland New Zealand; ^7^ School of Food and Advanced Technology College of Sciences, Massey University Palmerston North New Zealand

**Keywords:** DISEASES AND DISORDERS OF/RELATED TO BONE, MENOPAUSE, OSTEOPOROSIS, OTHER THERAPEUTICS

## Abstract

Osteoporosis and its precursor osteopenia are common metabolic bone diseases in postmenopausal women. A growing body of evidence suggests that the gut microbiota is involved in the regulation of bone metabolism; however, there are few studies examining how gut microbiomes in osteoporosis and osteopenia may differ from those in healthy individuals. The aim of this study was to characterize the diversity, composition, and functional gene potential of the gut microbiota of healthy, osteopenic, and osteoporotic women. Body composition, bone density, and fecal metagenomes were analyzed in 86 postmenopausal women. The women were classified as healthy, osteopenic, or osteoporotic based on *T*‐scores. The taxonomic and functional gene compositions of the microbiome were analyzed using shotgun metagenomic sequencing. Both osteoporotic and osteopenic taxonomic compositions were found to be significantly different from healthy participants. Linear discriminant‐analysis effect‐size analyses identified that healthy participants had more unclassified *Clostridia* and methanogenic archaea (*Methanobacteriaceae*) than in both osteoporotic and osteopenic participants. *Bacteroides* was found to be more abundant in osteoporosis and osteopenia groups. Some KEGG pathways, including carbohydrate metabolism, biosynthesis of secondary metabolites, and cyanoamino acid metabolism, were found to be more abundant in both osteoporosis and osteopenia. These results show that osteoporosis and osteopenia alter the gut microbiome of postmenopausal women and identify potential microbial taxonomic and functional pathways that may be involved in this disease. © 2020 The Authors. *JBMR Plus* published by Wiley Periodicals LLC. on behalf of American Society for Bone and Mineral Research.

## Introduction

Osteoporosis and its precursor osteopenia are metabolic bone diseases that are characterized by a reduction in bone mass and increased likelihood of fracture. In postmenopausal women, it is typically linked to estrogen deficiency, and its occurrence increases with age with an estimated 200‐million women affected worldwide.^(^ ^1^ ^)^ Throughout life, bones constantly undergo remodeling through cycles of resorption by osteoclasts and formation by osteoblasts, but bone integrity is lost and can lead to osteoporosis when resorption activities are greater than formation.^(^ ^2^ ^)^ The regulation of bone remodeling is influenced by a number of factors, including endocrine hormones and inflammatory cytokines.^(^ ^3^, ^4^, ^5^ ^)^

In recent years, it has become increasingly apparent that homeostasis of the gut microbiome is not only essential for maintaining human health but also contributes or is responsive to health conditions unrelated to the intestine.^(^ ^6^, ^7^, ^8^ ^)^ A growing body of evidence supports that the gut microbiota is linked to bone metabolic function and a range of bone diseases.^(^ ^9^, ^10^ ^)^ Experiments have shown that germ‐free mice have increased trabecular BMD than those conventionally raised.^(^ ^11^ ^)^ When germ‐free mice were recolonized with conventional gut microbes, BMD decreased, suggesting the gut microbiome is involved in bone mass regulation.^(^ ^11^ ^)^ Another colonization study in germ‐free mice found that donor age and nutritional condition appeared to play a role in how the gut microbiota influenced bone development.^(^ ^12^ ^)^ Several studies have shown that the use of prebiotics and/or probiotics can limit or prevent bone loss.^(^ ^13^, ^14^, ^15^, ^16^ ^)^ Although the gut microbiome appears to be linked to bone health, there is still limited data on how gut microbial composition and function may differ in those with metabolic bone disease.

Relatively few studies have investigated the composition of the gut microbiota in osteoporosis and/or osteopenia, and all previously published studies have used 16S rRNA analysis.^(^ ^17^, ^18^, ^19^ ^)^ These studies have also examined a mixture of men and women where their osteoporosis may have a more diverse and gender‐specific set of contributions to bone loss and microbiota composition. Gender has been shown to affect the composition and response of the gut microbiome.^(^ ^20^, ^21^, ^22^ ^)^ In this study, we focused on postmenopausal women. We used shotgun metagenomic sequencing to examine the differences in diversity, composition, and functional gene potential of the gut microbiota in healthy, osteopenic, and osteoporotic subjects. We hypothesized that women with osteoporosis and osteopenia would have an altered gut microbiome compared with healthy women. A previous study showed some evidence that there are differences in gut microbiota between osteoporotic and osteopenic patients,^(^ ^19^ ^)^ so they were investigated here in addition to comparisons with healthy subjects.

## Participants and Methods

### Subjects

Eighty‐six postmenopausal women aged 54 to 81 years participated in phase II of the “Bugs'n'Bones” study that took place in the Human Nutrition Unit of Massey University, Palmerston North between October 17, 2017 and March 6, 2018. The inclusion criteria required menopause of at least 5 years based on no menstruation. Exclusion criteria included the presence of any systemic disease, food intolerances that affect the gut, smokers, high intake of alcohol (>2 units/day), or use of antibiotics within 3 months of the study. Participants with significant weight loss or weight gain within the past year were also excluded. No participants were undergoing medical treatment for osteoporosis or osteopenia. Written informed consent was obtained from participants before commencing data collection. The study was registered with the Australian New Zealand Clinical Trials Registry (ANZCTR; ACTRN12617000802303) and was approved by Massey University Human Ethics Committee (Southern A, Application 17/17).

### Anthropometric and body composition measurements

Body composition measurements, fat mass, lean mass, and fat percentage were measured and analyzed using the Hologic QDR series Discovery A Bone Densitometry System (DXA). BMD was measured at the femoral neck (FN), lumbar spine (LS; L1‐L4), trochanter, Ward's triangle, and total hip. The DXA machine was calibrated every morning and at the end of the day for all measurements. Apex System Software version 4.5.3 was used to analyze the DXA scans. Osteoporosis was defined as *T*‐score ≤ 2.5 at the FN or LS and osteopenia as *T*‐score between −1.0 and −2.5 according to the WHO criteria. Twenty‐six women were classified as healthy, 42 as osteopenic, and 18 as osteoporotic based on WHO classifications, which diagnose using osteoporosis and osteopenia level *T*‐scores in either the LS or FN. Statistical analyses of participant data were performed using one‐way ANOVA tests (p < 0.05) and post‐hoc Tukey tests (p < 0.05). Normal distribution was confirmed by Levene's test of homogeneity of variance.

### Sample collection and DNA isolation

Participants were provided with a fecal sample collection kit and were instructed to collect feces into a container in an anaerobic bag with an anaerobic sachet and freezer pack and bring to the Human Nutrition unit within 2 to 3 hours of collection according to standard practice.^(^ ^23^ ^)^ Fecal samples were then stored at –80°C.

DNA was extracted from the fecal samples using the Bioline Isolate Fecal DNA kit as per the manufacturer's instruction within 1 week of sample collection and stored at –80°C before sequencing. The purity and concentration of DNA samples were tested using a Nanodrop 2000 Spectrophotometer (Thermo Fisher Scientific).

### Library preparation, shotgun metagenomic sequencing, and data analysis

The library preparation was conducted by Massey Genome Service using the Nextera XT DNA Library Prep kit (Illumina). DNA was then sent to Otago Genomics and Bioinformatics Facility for shotgun genomic sequencing using the HiSeq2500 System (Illumina).

Quality control of sequencing data was performed by KneadData^(^ ^24^ ^)^ using the paired‐end mode. Contaminants and human reads were filtered out by aligning to human reference genomes (hg37 and hg38) and contaminant and mitochondria databases available from KneadData. Only reads with both pairs passing quality control were retained for further analysis. The forward and reverse reads were then concatenated into a single file using Microbiome Helper's concat_paired_end.pl with flags (−n).^(^ ^25^ ^)^

Reads following quality control and concatenation were classified using DIAMOND^(^ ^26^ ^)^ blasting against the National Center for Biotechnology Information nr database (November 23, 2018). Output Direct Access Archive (DAA) files were analyzed in MEGAN Ultimate Edition,^(^ ^27^ ^)^ using absolute counts with the taxonomic and KEGG databases. Classifications and abundance files were exported from MEGAN in biom format and converted using the biom conversion tool^(^ ^28^ ^)^ to adjust classifications into the correct format. The sample groups were analyzed in a pairwise manner (healthy [H] vs osteoporosis [OP], healthy [H] vs osteopenia [OPN], osteoporosis [OP] vs osteopenia [OPN]). The abbreviated versions only refer specifically to our study groups. Calypso^(^ ^29^ ^)^ was used to analyze and visualize taxonomic data. Taxonomic data were filtered to remove reads below 0.05% (relative abundance) and normalized using total sum normalization (TSS). Alpha diversity was measured using the Shannon and Simpson diversity indices (Kruskal‐Wallis, *p* < 0.05). Beta diversity was measured using Bray‐Curtis principal coordinate analysis (PCoA) and a permutational multivariate analysis of variance (PERMANOVA) for statistical analysis (*p* < 0.05). Permutational analysis of multivariate dispersions (PERMDISP) was performed to measure the homogeneity of group dispersions in complement to the PERMANOVA analysis. Linear discriminant‐analysis effect‐size (LEfSe) analysis was performed to determine which taxa were significantly different between groups (linear discriminant analysis [LDA] ≥ 3, *p* < 0.05), and significant taxa were further subjected to a one‐way ANOVA (*p* < 0.05) as a secondary confirmation of results. MicrobiomeAnalyst^(^ ^29^ ^)^ was used to analyze KEGG functional data. KEGG functional data were filtered to remove reads below an abundance of 25 in 20% of the samples and low variance with a 10% interquantile range and normalized using TSS. LEfSe analysis identified significant differences in KEGG gene abundance between groups (LDA ≥ 3, false discovery rate [FDR] *p* value < 0.05). An LDA ≥ 3 was selected for both the taxonomic and functional analyses to be more stringent in the identification of relevant group differences.

## Results

### Study population

In this study, samples and clinical information from 86 postmenopausal women were analyzed (Table 1). Their ages ranged from 54 to 81 years, and they were classified as OP (n = 18), OPN (n = 42), or H (n = 26) based on their FN and LS *T*‐scores according to WHO classification. *T*‐scores and BMD values for H, OPN, and OP women were significantly different for LS, FN, and hip (ANOVA, *p* < 0.05). Although differences in BMI and waist circumference were significant between groups in this study, percentage body fat, body lean/fat mass ratio, and waist:hip ratio were not (ANOVA, *p* < 0.05).

**Table 1:** Study Participants' Data

Participant data	Healthy	OP	OPN	*p* Value
Age	62.5 ± 4.54	64.6 ± 5.66	63 ± 4.10	0.43
Body Mass Index	27 ± 2.96^a^	23.8 ± 3.72^b^	25.7 ± 4.01^ab^	**0.02**
Percentage Body Fat	40.6 ± 4.84	38.7 ± 8.23	40.8 ± 5.59	0.67
Body Fat/Lean Mass Ratio	0.69 ± 0.13	0.66 ± 0.2	0.70 ± 0.15	0.68
Waist Circumference	84.1 ± 8.04^a^	77.3 ± 12.13^b^	78 ± 11.3^a^	**0.03**
Waist‐Hip ratio	0.84 ± 0.07	0.80 ± 0.09	0.80 ± 0.07	0.13
Spine *T*‐score	0.47 ± 0.98^a^	−2.6 ± 0.93^b^	−1.48 ± 0.77^c^	**<0.00001**
Spine BMD	1.09 ± 0.1^a^	0.76 ± 0.1^b^	0.88 ± 0.09^c^	**<0.00001**
Total Hip *T*‐score	0.09 ± 0.64^a^	−1.92 ± 0.4^b^	−0.91 ± 0.67^c^	**<0.00001**
Total Hip BMD	0.95 ± 0.079^a^	0.71 ± 0.05^b^	0.82 ± 0.07^c^	**<0.00001**
Femoral Neck *T*‐score	−0.38 ± 0.47^a^	−2.4 ± 0.39^b^	−1.54 ± 0.42^c^	**<0.00001**
Femoral Neck BMD	0.8 ± 0.05^a^	0.59 ± 0.05^b^	0.67 ± 0.05^c^	**<0.00001**

The values represent the mean ± SD for each group. One‐way ANOVA tests were used to determine statistical significance between groups (p < 0.05) and post‐hoc Tukey tests indicated pairwise differences (p < 0.05).

### Microbial community diversity and taxonomic composition

**Fig 1:**
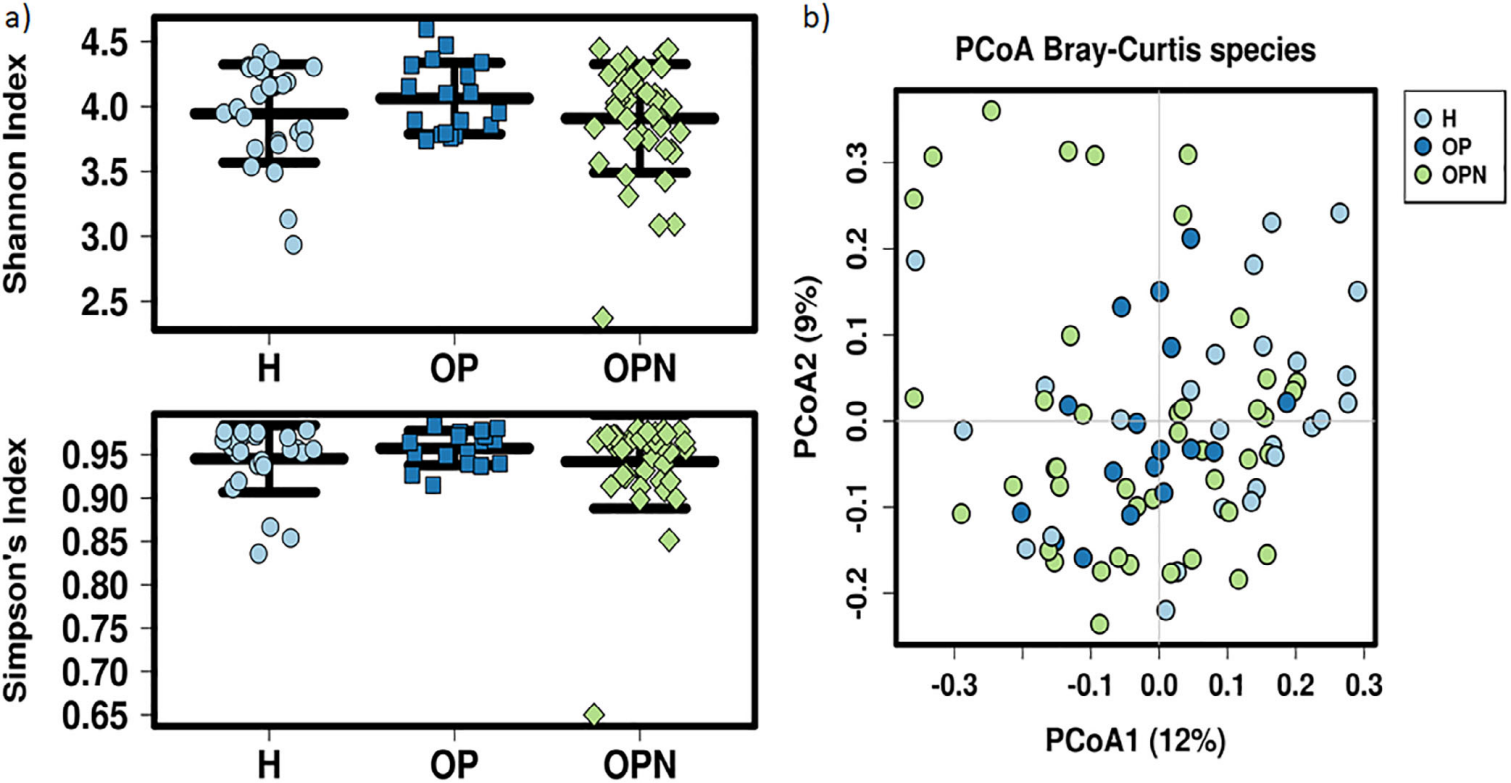
Alpha and beta diversity plots to examine the differences between participant groups. (*A*) Shannon and Simpson index diversity plots for healthy (H), osteoporosis (OP), and osteopenia (OPN) group participants. (*B*) Bray‐Curtis principal coordinate analysis (PCoA) plot of the H, OP, and OPN group participants.

To determine if there were differences between groups, microbial diversity and composition were examined. Direct comparisons were done in a pairwise manner (H vs OPN, H vs OP, OP vs OPN). The Shannon and Simpson indices were used to determine alpha diversity. No significant difference was observed between any of the groups (Kruskal‐Wallis *p* > 0.05; Fig. 1A). To examine beta diversity, a Bray‐Curtis principal coordinate analysis (PCoA) was performed. The PCoA plot did not show an obvious separation between groups (Fig. 1*B*). To determine if there were significant differences between the groups, PERMANOVA was performed. Comparisons of H versus OP (*p* = 0.010) and H versus OPN (*p* = 0.048) groups indicated significant differences, whereas OP versus OPN (*p* = 0.628) groups did not. To look at the potential influence of intragroup variation on the PERMANOVA analysis, PERMDISP was performed (Supplementary Fig. S1). The results were not statistically significant, indicating the PERMANOVA differences were not caused by greater intragroup variability.

### Identification of significant taxa differences

**Fig 2:**
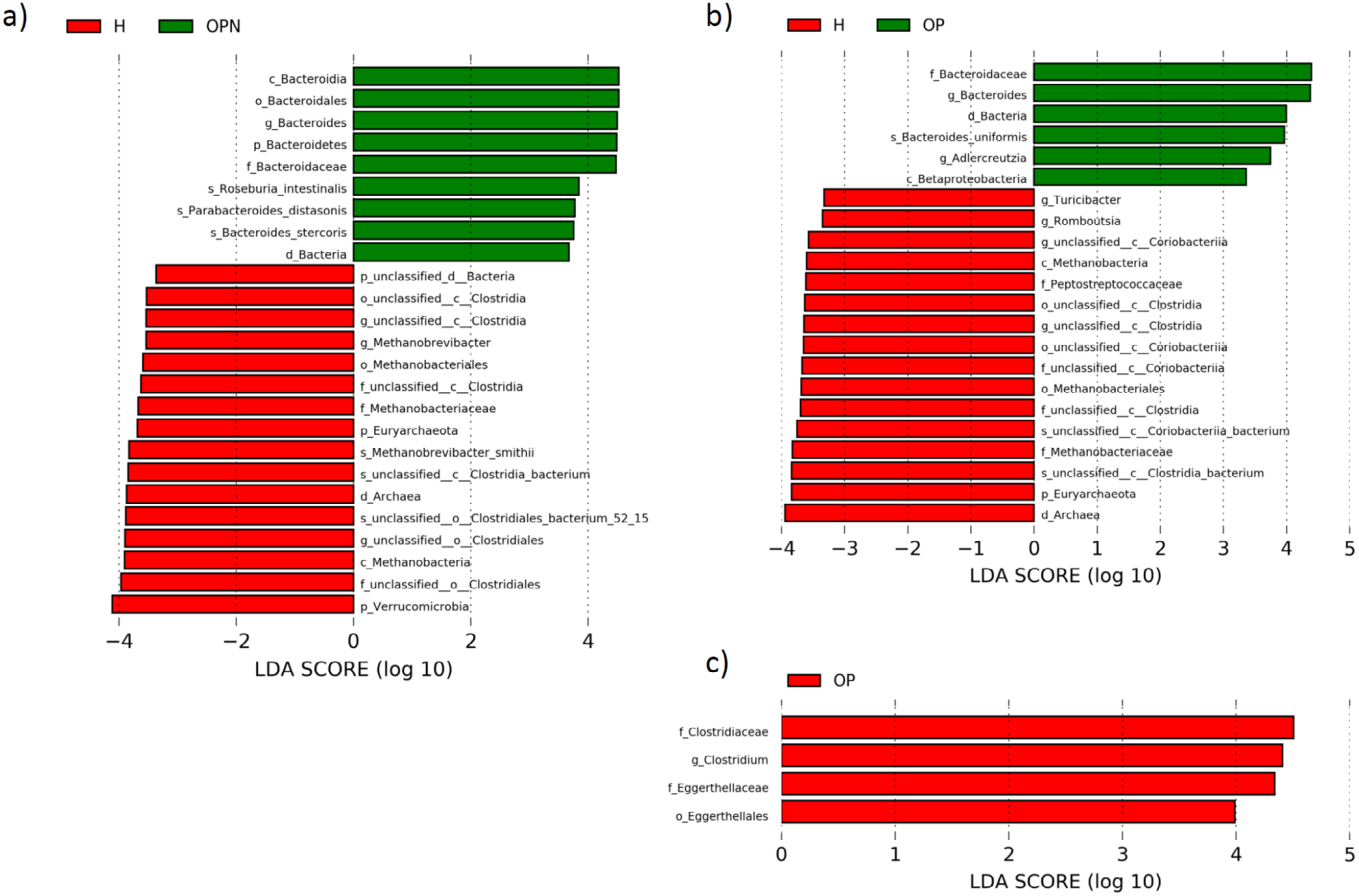
Linear discriminant analysis effect size (LEfSe) analyses used to identify the taxonomic features most likely to explain the differences between groups. (*A*) Pairwise taxonomic LEfSe analysis of healthy (H) and osteopenia (OPN) groups (linear discriminant analysis [LDA] ≥ 3, *p* values < 0.05). (*B*) Pairwise taxonomic LEfSe analysis of H and OP groups (LDA ≥ 3, *p* values < 0.05). (*C*) Pairwise taxonomic LEfSe analysis of OP and OPN groups (LDA ≥ 3, *p* values < 0.05).

To identify meaningful differences in specific taxonomic groups, LEfSe was used to identity taxa with significant differences between groups (LDA ≥ 3; *p* value < 0.05). Results were examined further and visualized using ANOVA plots (*p* value < 0.05; Supplementary Figs. S2‐S4). A total of 22, 25, and 4 taxa were identified as significant for the H versus OP, H versus OPN, and OP versus OPN comparisons (Fig. 2*A*‐*C*) ranging from domain to species classification. Approximately half of the significant taxa were shared between the H versus OP and H versus OPN comparisons. Taxa more abundant in both OP and OPN groups were primarily *Bacteroides*, whereas those higher in abundance in the H group in both comparisons were unclassified *Clostridia* and *Methanobacteriaceae* (eg, *Methanobrevibacter smithii*). The OPN group also had a greater abundance of *Parabacteroides distasonis*, *Bacteroides uniformis*, and *Roseburia intestinalis*, whereas its comparative H group had higher Verrucomicrobia and unclassified *Clostridiales* bacterium 52_15. OP women had higher numbers of Betaproteobacteria, *Bacteroides stercoris*, and *Adlercreutzia*, whereas the corresponding H women had greater amounts of *Peptostreptococcaceae*, *Turicibacter*, *Romboutsia*, and unclassified *Coriobacteriia* bacterium. For the OP and OPN comparison, the OP group was found to have greater numbers of *Eggerthellaceae* and *Clostridium*.

### Microbial functional metagenome analysis

To identify potential functional differences between groups, the shotgun metagenome data were mapped to the KEGG database. LEfSe analysis was used to identify significantly different clusters and pathways from KEGG level 2 to level 4 (Table 2). Like the taxonomic comparisons, several identified pathways were shared by the OP and OPN groups as compared with the H group. Carbohydrate metabolism, biosynthesis of other secondary metabolites, environmental adaptation, phenylpropanoid biosynthesis, and cyanoamino acid metabolism were higher in OP and OPN groups as compared with the H group. For the H versus OPN comparison, replication and repair pathways were higher in the H group. In contrast, for H versus OP groups, there were more abundant unclassified signaling and cellular processes, biotin metabolism, and pentose and glucuronate interconversions in the OP group. There were no differences in functional gene abundances between OP and OPN groups.

**Table 2:** Pairwise KEGG‐Based LEfSe Analysis of Healthy Versus Osteopenia and Healthy Versus Osteoporosis Groups (LDA ≥ 3, FDR *p* value < 0.05) to Identify the Features Most Likely to Explain the Differences Between Groups

**H vs OPN**	**H**	**OPN**	***p* Value**	**FDR**	**LDA**
Environmental adaptation	24572	**26674**	0.0011	0.0217	−3.02
Biosynthesis of other secondary metabolites	131890	**139660**	0.0012	0.0217	−3.59
Replication and repair	**249360**	238420	0.0022	0.0217	3.74
Carbohydrate metabolism	833440	**854070**	0.0023	0.0217	−4.01
ko00460 Cyanoamino acid metabolism	29885	**34730**	0.0002	0.0406	−3.38
ko00940 Phenylpropanoid biosynthesis	19645	**24432**	0.0003	0.0406	−3.38
**H vs OP**	**H**	**OP**	***p* Value**	**FDR**	**LDA**
Biosynthesis of other secondary metabolites	131890	**142200**	0.0012	0.0294	−3.71
Environmental adaptation	24572	**27610**	0.0016	0.0294	−3.18
Carbohydrate metabolism	833440	**859650**	0.0019	0.0294	−4.12
Unclassified: signaling and cellular processes	145400	**150600**	0.0039	0.0445	−3.41
ko00780 Biotin metabolism	21610	**23844**	0.0001	0.0115	−3.05
ko00940 Phenylpropanoid biosynthesis	19645	**25721**	0.0001	0.0115	−3.48
ko00040 Pentose and glucuronate interconversions	40646	**45251**	0.0001	0.0115	−3.36
ko00460 Cyanoamino acid metabolism	29885	**35872**	0.0002	0.0115	−3.48

FDR = false discovery rate; H = healthy; LDA = linear discriminant analysis; OP = osteoporosis; OPN = osteopenia.

## Discussion

This is the first published study to use shotgun metagenomic sequencing to examine osteoporotic and osteopenic gut microbiomes specifically in postmenopausal women. We identified that OP and OPN microbial communities were significantly different from H communities but not each other. However, there was no difference in Shannon or Simpson diversity between any of the groups. This matches with previous studies using 16S rRNA sequencing that identified community differences between osteoporotic and healthy cohorts but did not observe differences in Shannon or Simpson diversity.^(^ ^17^, ^19^ ^)^ One study did identify differences in alpha diversity between healthy, osteoporotic, and osteopenic subjects, but there were only six participants per group and should be interpreted carefully.^(^ ^18^ ^)^

Investigation into individual taxa found that the H versus OP and H versus OPN analyses revealed many similarities with *Bacteroides* associated with OP and OPN groups and unclassified *Clostridia* and methanogenic archaea associated with the H group. The functional analysis supported this observation of similarities, suggesting the possibility that there is a directional shift in the gut microbiome that starts with osteopenia and remains similar during osteoporosis. A previous study supported that osteoporotic and osteopenic bacterial communities were much more similar to each other than healthy communities.^(^ ^18^ ^)^

Our observations of increased *Bacteroides* along with classifications up to the Bacteroidetes phylum in the OP group matched with results from Xu et al but contrasted with an observed lower abundance in Bacteroidetes in the Wang et al study.^(^ ^17^, ^18^ ^)^ This difference may be because of the lower analysis stringency in the Wang study, along with its low number of participants. *Erysipelotrichaceae*, of which *Turicibacter* is a member, has also previously been shown to be higher in healthy subjects compared with osteoporotic.^(^ ^17^ ^)^ The lack of information on the potential role of archaea in previous studies on these conditions is not surprising. The primers that are typically used for 16S high‐throughput sequencing are not ideal for the amplification of archaea, and many analysis pipelines specifically filter these sequences out.^(^ ^30^, ^31^ ^)^

Members of the *Clostridia*/*Clostridiales* are made up of a diverse group of organisms. A number of families and genera within these groups have been identified as significantly different in previous studies on osteoporosis,^(^ ^17^, ^19^ ^)^ but it is not as clear cut as broad taxonomic classifications associating specifically with healthy, osteoporotic, or osteopenic groups. This finding was observable in our study as the species *Roseburia intestinalis* was more abundant in the OPN group versus H group, whereas unclassified members of *Clostridia* and *Clostridiales* down to the genera or species level were more abundant in the H group compared with OP and OPN participants. *Rombousia* (*Peptostreptococcaceae*), a *Clostridia* member, was also identified as higher in H versus OP participants.

**Fig 3:**
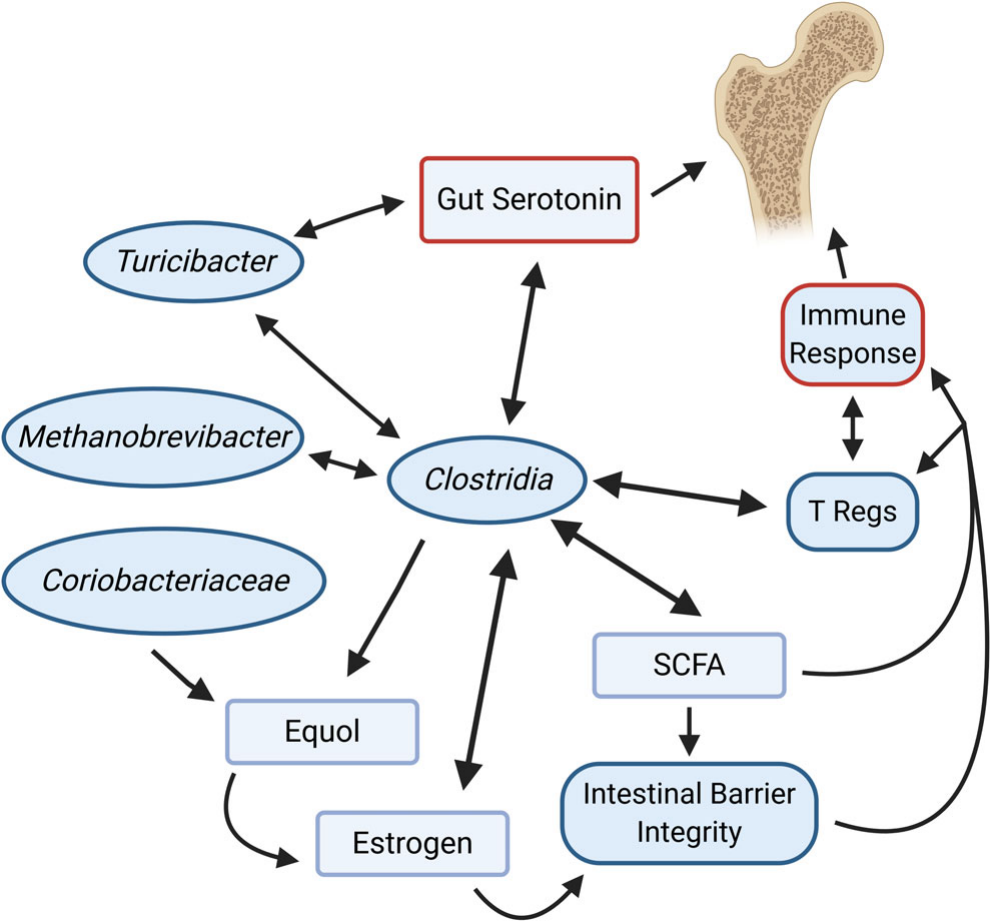
Potential mechanisms by which microbes identified in this study and particular members of *Clostridia*, may influence bone metabolism. These include (i) direct and indirect impacts on the immune system via T regulatory cell activation, hormones, and intestinal integrity, (ii) co‐occurrence relationships between microbes, and (iii) microbial regulation of gut serotonin. (Created with BioRender.com). SCFA = short‐chain fatty acid.

It is interesting to observe the reoccurring appearance of *Clostridia* members, along with other taxa observed in our study that have direct or indirect links to previously published studies on bone health (Fig. 3). In a clinical study, the abundance of nonovarian estrogens in postmenopausal women and men was correlated with four *Clostridia* taxa.^(^ ^32^ ^)^

Isoflavones are plant‐based micronutrients with weakly oestrogenic activity typically found in soy‐based products.^(^ ^33^ ^)^ These isoflavones can be metabolized to equol, which has a stronger affinity for the estrogen receptor‐beta,^(^ ^34^, ^35^ ^)^ by intestinal microorganisms including *Clostridia* and *Coriobacteriaceae*.^(^ ^33^, ^36^, ^37^ ^)^ Treatment with isoflavones or equol has been shown to reduce bone loss in both rodent models and postmenopausal women.^(^ ^38^, ^39^, ^40^ ^)^ The presence of isoflavones has also been shown to decrease the abundance of *Bacteroides* and *Parabacteroides*.^(^ ^33^ ^)^ These two genera were associated with osteoporosis and osteopenia in our study. *Veillonella*, another known equol producer, was previously identified as significantly more abundant in healthy subjects than osteoporotic and higher in osteopenic than osteoporotic, but not different between healthy and osteopenic.^(^ ^19^ ^)^

Estrogen is known to play a role in the regulation of the intestinal epithelial barrier, and a deficiency can lead to increased permeability.^(^ ^9^, ^41^ ^)^ A decrease in intestinal permeability caused by estrogen deficiency triggers inflammatory responses and promotes osteoclast formation, which can lead to bone loss.^(^ ^9^ ^)^ Alternatively, sufficient estrogen can activate regulatory T cells (Tregs), which prevent osteoclastogenesis and osteoblast formation to improve/maintain bone mass.^(^ ^9^, ^42^, ^43^ ^)^ Tregs have also been shown to be induced by a mixture of human *Clostridia* strains.^(^ ^44^ ^)^

*Clostridia* are also well‐known producers of short‐chain fatty acids (SCFAs), including butyrate, which has been shown to enhance the gut epithelial barrier^(^ ^45^ ^)^ and can induce colonic regulatory T cells.^(^ ^46^ ^)^ Lucas et al showed that treating mice with SCFA or feeding a high‐fiber diet prevented postmenopausal and inflammatory bone loss and significantly increased bone mass.^(^ ^47^ ^)^ Tregs increased after SCFA treatment, and it was specifically C3 (propionate) and C4 (butyrate) SCFAs that were shown to regulate osteoclast metabolism.^(^ ^47^ ^)^ Another common byproduct of microbial fermentation in the colon is hydrogen (H_2_). A number of *Clostridia* members are known H_2_ producers with some strains reported to produce considerable amounts in vitro.^(^ ^48^, ^49^, ^50^ ^)^ We observed an increased abundance in certain *Clostridia* members and methanogens in H participants as compared with both those with OPN and OP. In a twin study, methanogen abundance (ie, *Methanobrevibacter smithii*) was positively correlated to 20 species belonging to *Clostridiales*, suggesting a H_2_‐based relationship.^(^ ^51^ ^)^

Osteoblasts, which are involved in bone formation, are known to have serotonin receptors.^(^ ^52^, ^53^ ^)^ Although there is much controversy in whether gut‐derived serotonin plays a positive or negative role in bone strength,^(^ ^54^, ^55^, ^56^, ^57^ ^)^ there is some evidence that differences in its concentration could cause opposite effects on osteoblasts.^(^ ^58^ ^)^ This finding suggests that regulation of gut‐derived serotonin concentrations may influence bone remodeling. Several studies have linked *Clostridia* along with *Turicibacter* to gut serotonin regulation.^(^ ^59^, ^60^ ^)^ Fung et al demonstrated that orally supplying serotonin to specific pathogen‐free (SPF) mice substantially increased *Clostridia* abundance.^(^ ^60^ ^)^ Experiments in serotonin‐transporter–deficient mice, which have modestly increased host‐derived intestinal serotonin, enriched for *Clostridia* (*Clostridiaceae*) and *Turicibacter*; however, use of fluoxetine, a selective serotonin reuptake inhibitor (SSRI), in SPF mice caused a decrease in *Turicibacter* and *Clostridiaceae* abundance without a significant change in fecal serotonin content.^(^ ^60^ ^)^ A study in human twins, found that SSRI use was negatively associated with *Turicibacteraceae*.^(^ ^61^ ^)^ The inability of these bacteria to uptake serotonin appears to drive their decrease in abundance. *Turicibacter sanguinis* has been shown to be able to import and use serotonin and *Turicibacter* abundance correlated with intestinal serotonin levels.^(^ ^60^ ^)^ In a previous study, *Turicibacter* and *Clostridia* species were shown to be part of consortium able to regulate serotonin biosynthesis, whereas all tested *Bacteroides* species did not affect peripheral host serotonin.^(^ ^59^ ^)^ The levels of *Turicibacter* in our study do not necessarily suggest an increased presence of gut serotonin in the H subjects as they were similar to untreated mice,^(^ ^60^ ^)^ but the lower abundance in OP subjects may indicate a reduced regulation of gut‐derived serotonin, which may in turn have an influence on bone remodeling.

The majority of functional genes identified as differentially abundant in this study were higher in OP and OPN participants. Only one KEGG functional cluster, replication and repair, was more abundant in the H women, and it was only significant when compared with OPN women. Although there were many similarities identified in the H versus OPN and H versus OP comparisons, unclassified signaling and cellular processes, biotin metabolism, and pentose and glucoronate interconversions were additionally identified as more abundant in OP participants.

Environmental adaptation was identified as significantly more abundant for both the OP and OPN groups. It is unlikely that this is relevant to this study as this KEGG pathway relates to eukaryotic organismal system pathways tied to circadian rhythms, thermogenesis, and plant‐pathogen interactions.^(^ ^62^ ^)^ There may be microbial gene homologs, but it seems more likely that there is sequence similarity without functional similarity.

The increase in gene abundance of the biosynthesis of other secondary metabolites' cluster in OP and OPN participants may suggest the increased presence of genes coding for biosynthesis of antimicrobial compounds, which could give carriers a competitive advantage against susceptible bacteria.^(^ ^62^ ^)^ Phenylpropanoid biosynthesis falls under this category. Phenylpropanoids are typically associated with plants,^(^ ^63^ ^)^ but bacterial homologs exist and have been linked to antimicrobial synthesis pathways.^(^ ^64^ ^)^

The increased abundance in carbohydrate metabolism for OP and OPN participants may be linked to the increased abundance of *Bacteroides* in those groups. *Bacteroides* species are highly adaptive polysaccharide users that carry an array of diverse enzymes to break down sugar molecules.^(^ ^65^ ^)^ They are able to fill a variety of niches where more specialized microbes may struggle under challenging nutritional conditions.^(^ ^65^ ^)^ Bacteriodetes may also be more efficient at extracting energy from diet from lean individuals, which could contribute to increased abundance in osteoporosis patients as low body mass index is a risk factor.^(^ ^66^, ^67^ ^)^

Interestingly, in a study of human osteoclasts, cyanoamino acid metabolism expression was downregulated in samples treated with bisphosphonates, drugs commonly used to treat osteoporosis by inhibiting bone resorption.^(^ ^68^ ^)^ In a glucocorticoid‐induced osteoporosis rat model, cyanoamino acid metabolism was identified as a potential biomarker for osteoporosis using metabolomics.^(^ ^69^ ^)^ This finding aligns with our results in which cyanoamino acid metabolism was higher in OP and OPN participants. However, it should be kept in mind that the results from previous studies came from a eukaryotic host or its tissues, rather than the microbiome and may be unrelated to the results seen from this study. Alterations in this pathway from microbes may affect the host organism in similar ways; any connections should be treated with caution.

Shotgun metagenomic sequencing data are useful for taxonomic identification and our understanding of the potential functionality of microbes, but still lack the evidence of gene activity. Future studies would be enhanced with the use of microbial RNA‐Seq and metabolome data alongside the integration of specific human biological data, including immune markers and hormone levels, to the analysis. A consideration of dietary habits and activity levels would also be ideal to control for any effects they may have on study results. While large and equal numbers of subjects would ideally fall into each tested group of such a study, it is often impractical. This limitation may give more statistical weight and power to particular groups to help identify connections and give stronger certainty in the analyses and could be why we observed more taxa with statistical significance in comparisons involving the OPN group. The uneven group numbers may have limited the discovery of further potential differences. It is also important to recognize that while our study suggests that osteoporosis and osteopenia lead to alterations in the gut microbiome, it still does not directly prove a causal link to these conditions.

Although there is mounting evidence that the gut microbiome is linked to bone metabolic function, the identification of specific microbes or what microbial functional capabilities may contribute is still unknown. The microbes identified in this study, along with the connections to previous research, support further investigation into the relationship between the gut microbiome and bone and provide potential avenues for the focus of future studies.

## Disclosures

The authors have nothing to disclose.

## AUTHOR CONTRIBUTIONS

**Elizabeth Rettedal:** Formal analysis; supervision; visualization; writing‐original draft; writing‐review and editing. **Bolaji Ilesanmi‐Oyelere:** Investigation; methodology; writing‐review and editing. **Nicole Roy:** Resources; supervision; writing‐review and editing. **Jane Coad:** Conceptualization; methodology; supervision; writing‐review and editing. **Marlena Kruger:** Conceptualization; funding acquisition; methodology; supervision; writing‐review and editing.

## Data Availability

The sequencing data have been submitted to the NCBI SRA database under accession number PRJNA672125.

## Supporting information


**Supplementary Figure S1** PERMDISP analysis for H versus OP and H versus OPN comparisons.
**Supplementary Figure S2.** ANOVA Plots for H versus OP comparison.
**Supplementary Figure S3.** ANOVA Plots for H versus OPN comparison.
**Supplementary Figure S4.** ANOVA Plots for OP versus OPN comparison.
