# Supplementary material for: The Gut Microbiome Is Altered in Postmenopausal Women With Osteoporosis and Osteopenia
Source: JBMR Plus. 2021 Jan 19;5(3):e10452. doi: 10.1002/jbm4.10452 (PMC7990138; doi:10.1002/jbm4.10452)
Supplement: Supplementary file 1 — Supplementary Figure S1 PERMDISP analysis for H versus OP and H versus OPN comparisons. Supplementary Figure S2. ANOVA Plots for H versus OP comparison. Supplementary Figure S3. ANOVA Plots for H versus OPN comparison. Supplementary Figure S4. ANOVA Plots for OP versus OPN comparison. [file JBM4-5-e10452-s001.pdf]

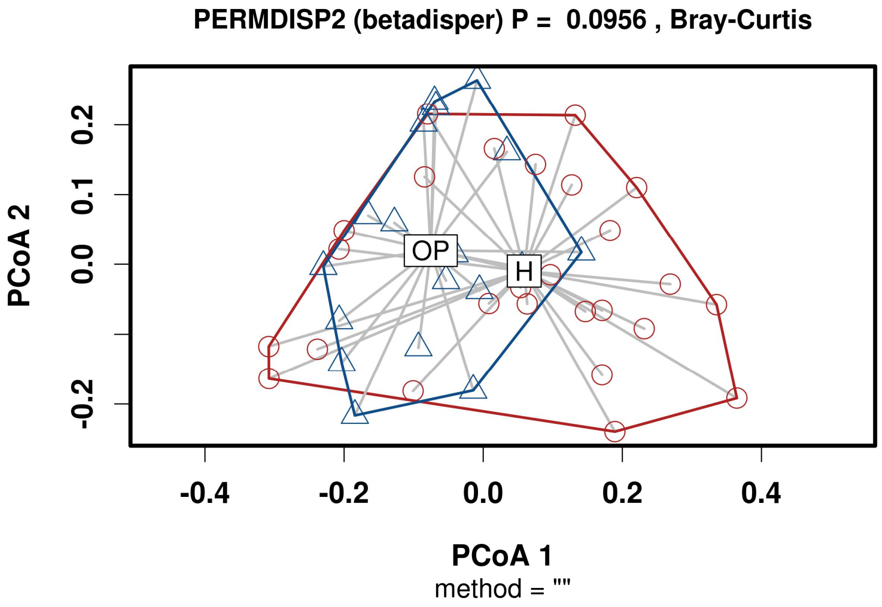

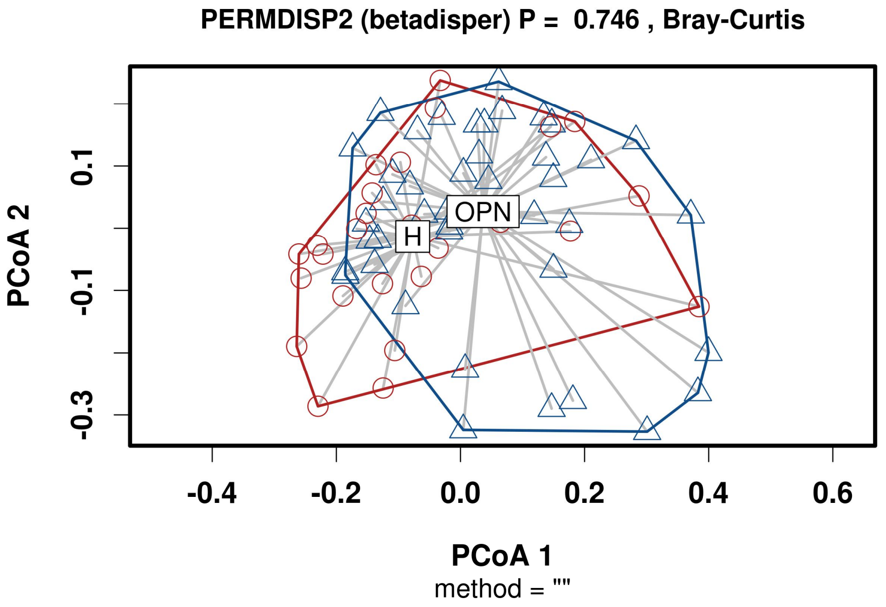

Figure S1. PERMDISP analysis for H vs OP and H vs OPN comparisons.

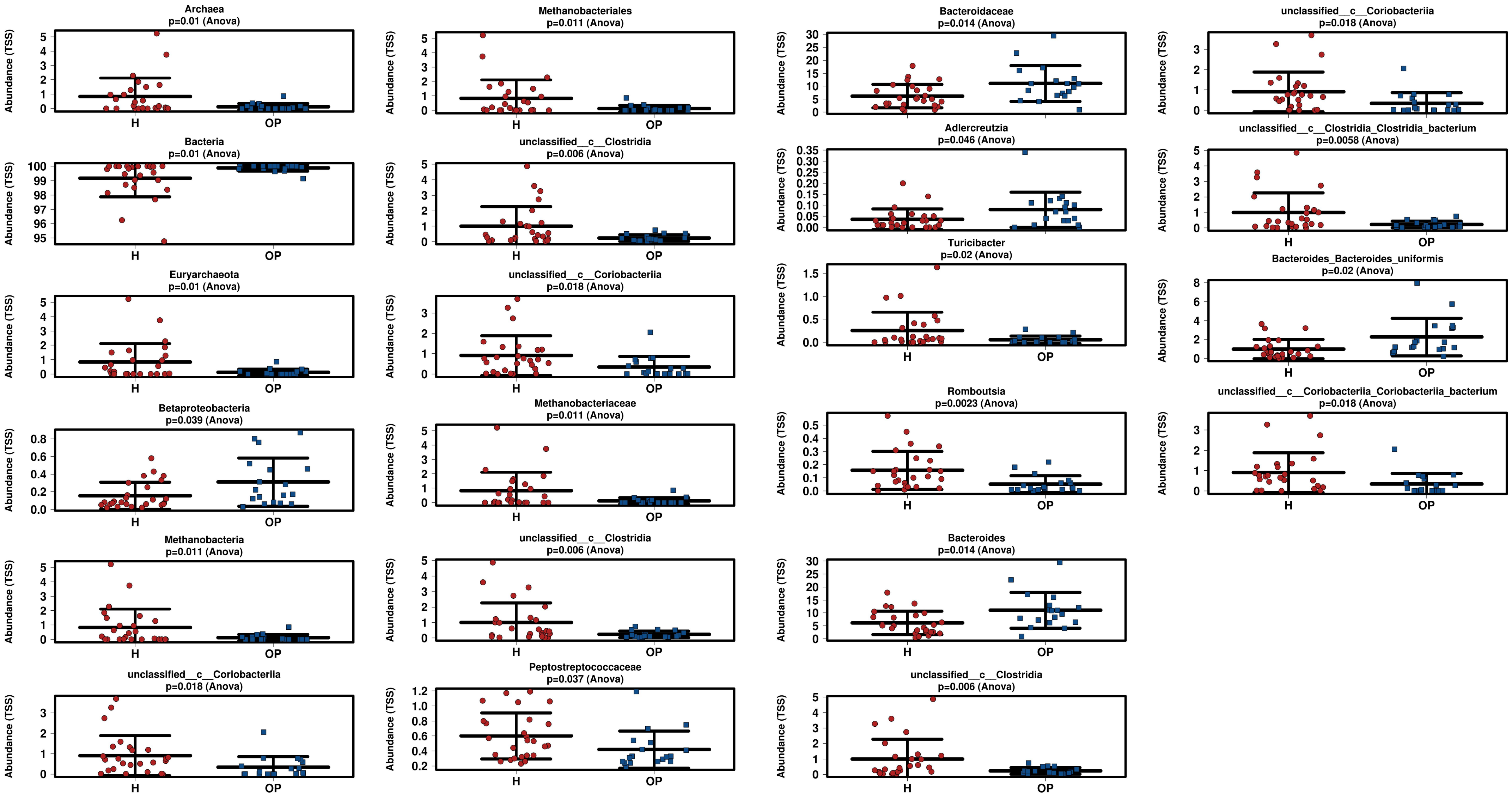

Figure S2. ANOVA Plots for H vs OP comparison.

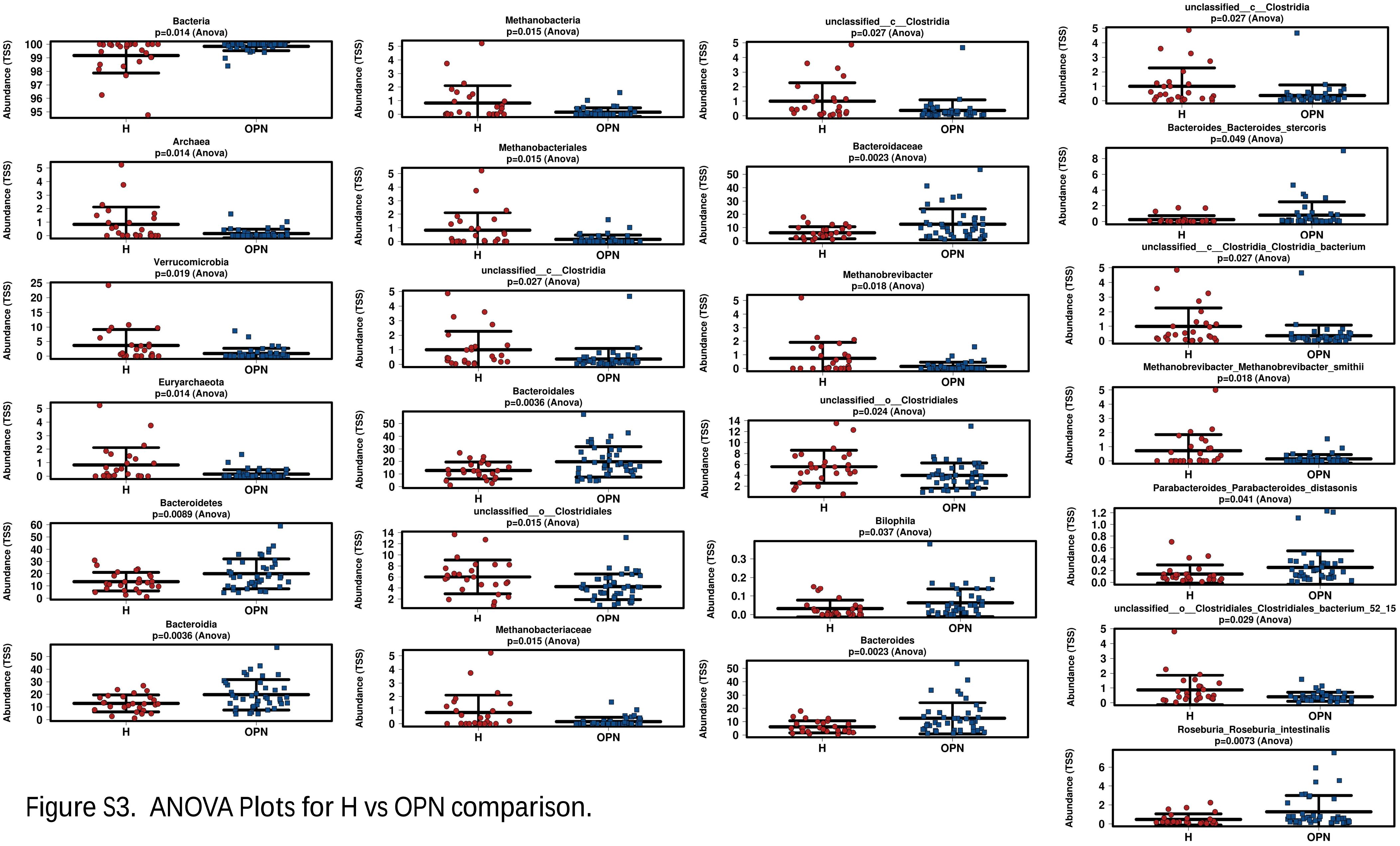

Figure S3. ANOVA Plots for H vs OPN comparison.

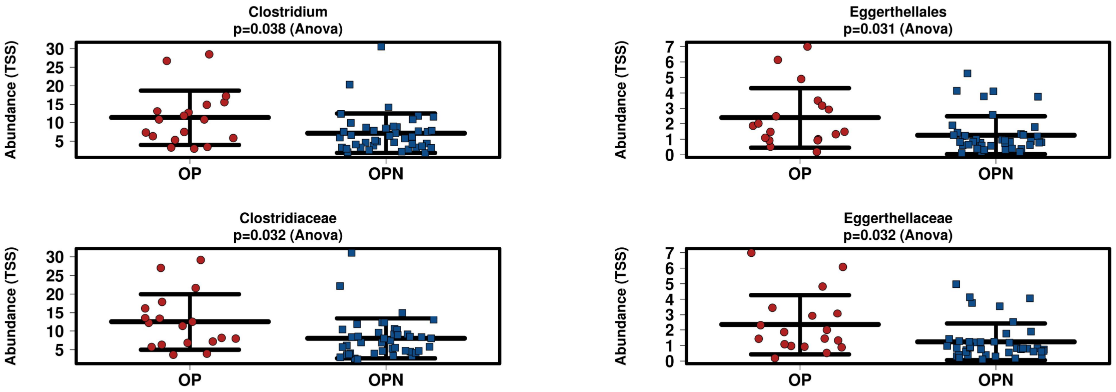

Figure S4. ANOVA Plots for OP vs OPN comparison.
